# Increased PD-1 Expression and Altered T Cell Repertoire Diversity Predict Mortality in Patients with Septic Shock: A Preliminary Study

**DOI:** 10.1371/journal.pone.0169653

**Published:** 2017-01-10

**Authors:** Atsutoshi Tomino, Masanobu Tsuda, Ruri Aoki, Yuka Kajita, Masamitsu Hashiba, Tsuguaki Terajima, Hideki Kano, Naoshi Takeyama

**Affiliations:** Department of Emergency and Critical Care Medicine, Aichi Medical University, Aichi, Japan; Universita degli Studi di Palermo, ITALY

## Abstract

Sepsis causes impairment of innate and adaptive immunity by multiple mechanisms, including depletion of immune effector cells and T cell exhaustion. Although lymphocyte dysfunction is associated with increased mortality and potential reactivation of latent viral infection in patients with septic shock, the relation between viral reactivation and lymphocyte dysfunction is obscure. The objectives of this study were 1) to determine the relation of lymphocyte dysfunction to viral reactivation and mortality, and 2) to evaluate recovery of lymphocyte function during septic shock, including T cell receptor (TCR) diversity and the expression of programmed death 1 (PD-1). In 18 patients with septic shock and latent cytomegalovirus (CMV) infection, serial blood samples were obtained on days 1, 3, and 7 after the onset of shock, and immune cell subsets and receptor expression were characterized by flow cytometry. TCR diversity of peripheral blood mononuclear cells was analyzed by Multi-N-plex PCR, and CMV DNA was quantified using a real-time PCR kit. A decrease of TCR diversity and monocyte HLA-DR expression were observed in the early stage of septic shock, while CD4+ T cells displayed an increase of PD-1 expression. Significant lymphopenia persisted for at least 7 days following the onset of septic shock. Normalization of TCR diversity and PD-1 expression was observed by day 7, except in patients who died. CMV reactivation was detected in 3 of the 18 patients during the first week of their ICU stay and all 3 patients died. These changes are consistent with the early stage of immune cell exhaustion and indicate the importance of normal lymphocyte function for recovery from septic shock. Ongoing lymphocyte dysfunction is associated with CMV reactivation and dissemination, as well as with unfavorable outcomes.

## Introduction

Sepsis is a complex syndrome that is thought to represent dysregulation of the host response to infection [1]. Both pathogen-related factors and host resistance factors contribute to the diversity of immune responses. In sepsis, immune function is suppressed, with a variety of consequences such as failure to control the primary infection, reactivation of latent viral infection, and development of secondary infection, often caused by microorganisms that are not particularly virulent for an immunocompetent host [2–7]. Thus, immune dysregulation is now considered to be a central event in the pathophysiology of sepsis [8, 9].

There is a growing body of evidence that sepsis is associated with impairment of both innate and adaptive immunity and may lead to the development of immune paralysis [3]. Mechanisms that suppress immunity include reduction of circulating lymphocytes due to increased apoptosis, decreased T cell proliferation and cytokine production in response to stimulation, and an increase of circulating regulatory T cells [2, 3, 8]. Such lymphocyte dysfunction is associated with increased mortality in patients with septic shock, as well as with the risk of reactivation and dissemination of latent viral infection [4, 5], but there has been no comprehensive investigation of the relation between viral reactivation and lymphocyte dysfunction in patients with sepsis.

Accordingly, the aims of this study were 1) to assess the relation of lymphocyte dysfunction to viral reactivation and mortality in patients with septic shock and latent cytomegalovirus (CMV) infection, and 2) to evaluate the recovery of lymphocyte function during sepsis, including changes in expression of the inhibitory receptor programmed death 1 (PD-1) and changes of T cell receptor (TCR) diversity.

## Materials and Methods

### Patients

This study was conducted in conformity with the declaration of Helsinki and was approved by the Institutional Review Boards of Fujita Health University and Aichi Medical University (#150 and #13–137). After receiving review board approval, written informed consent was obtained from each subject.

The study was performed in Japanese patients admitted to the ICU of our hospital during the 26-month period from October 2013 to November 2015. None of the subjects were related. Patients were screened for systemic inflammatory response syndrome (SIRS) and for organ dysfunction every day until discharge from ICU. SIRS was defined as being present if two or more of the diagnostic criteria of Bone et al. [10] were fulfilled. Organ failure was defined as a Sequential Organ Failure Assessment (SOFA) score ≥2 for the organ in question [11]. Septic shock was defined as hypotension (systolic BP ≤ 90 mmHg or decrease of systolic BP by ≥ 40 mmHg from baseline) persisting for at least 1 h despite adequate fluid infusion and requiring pressor agents to maintain a systolic BP around 90 mm Hg or mean arterial pressure <65 mmHg, associated with hypoperfusion (including lactic acidosis, oliguria, and/or acute changes of mental state) and documented infection [12]. Patients aged between 60 and 86 years old with septic shock and failure of at least one organ were eligible for the study. Patients with a terminal illness, patients who had been resuscitated after cardiac arrest, and patients admitted with acute or chronic liver failure (defined as the presence of any liver disease) were excluded. Blood was collected for analysis on the day when septic shock was diagnosed. The control subjects were 10 healthy age-matched volunteers with no significant acute or chronic illnesses.

The goal of treatment in the ICU was to maintain and restore vital functions. Mean arterial pressure was maintained at ≥65 mmHg, urine output at ≥0.5 ml/hr/kg, and central venous oxygen saturation (in the superior vena cava) at ≥70% or mixed venous saturation at ≥65%. Patients received empirical broad-spectrum antibiotic therapy based on the expected sensitivity profile of the probable pathogen. If a positive culture result was obtained, antibiotic therapy was tailored to the susceptibility of the pathogen thus identified. A specific focus of infection was identified within 6 hours of presentation and the most frequent site was the digestive tract. The 28-day mortality rate of the patients with septic shock was 17%.

### Blood samples

Patients provided consent for serial blood samples to be obtained on days 1, 3, and 7 after the onset of septic shock. The samples (10 ml) were collected in heparinized tubes, with 2 ml being used for FACS analysis and 6 ml for isolation of peripheral blood mononuclear cells (PBMCs). The remaining blood was centrifuged at 500 g for 5 min at 4°C to obtain plasma, which was frozen in small aliquots and stored at -80°C until assays were performed. After PBMCs were isolated by Ficoll-Hypaque density gradient separation using the standard protocol, genomic DNA was extracted by standard techniques to investigate TCR diversity.

### Analysis of T cell receptor diversity

Using genomic DNA isolated from PBMCs, TCR diversity was assessed with the Human Immun TraCkeRbeta test (ImmunID Technologies, Grenoble, France). Multi-N-plex PCR was performed with a specific primer for a V gene family and several specific primers for J segments, and the fluorescence intensity obtained was compared with that of the reference markers. Twenty-three different reactions were performed to cover all 276 possible T cell receptor betaV (TRBV)-T cell receptor betaJ (TRBJ) rearrangements. Rearrangements were detected and maps were generated and analyzed by using Constel’ID software (ImmunID Technologies). PCR was performed by using iProof enzyme (Bio-Rad) under the following conditions: 98°C for 3 min, 98°C for 20 sec, 72°C for 20 sec, and 72°C for 3.5 min. Cycles were performed with the annealing temperature being reduced by 1°C degree every cycle until it reached 68°C, after which 23 cycles were done at a constant temperature. The reaction was stopped at the exponential stage and final extension was performed for 10 min at 72°C. The actin gene was also amplified in each PCR as a control for normalization of DNA. PCR products (V-J1, J2, J3, J4, and Jn) were separated by size (with a maximum amplicon size of 5 kb) on 0.8% agarose gel, followed by direct staining with SYBR Green I and quantification using a CCD camera (Vibert Lourmart, France). Results were expressed as the percentage of rearrangements detected among the total of 276 possible combinatorial rearrangements, with a decreased percentage indicating a state of divpenia.

### Flow cytometric analysis

Antibodies were purchased from BD Pharmingen (San Diego, CA), eBiosciences (San Diego, CA), or Beckman Coulter (Indianapolis IN). For determination of PD-1 expression by CD4+ lymphocytes, 100 μl of heparinized whole blood was reacted directly with phycoerythrin (PE)-conjugated anti-PD-1 antibody (clone MIH4) and phycoerythrin/cyanin (PC5)-conjugated anti-CD4 antibody (clone 13B8.2) for 10 min at room temperature. For determination of human leukocyte antigen-DR (HLA-DR) expression by monocytes, 100 μl of heparinized whole blood was reacted directly with fluorescein isothiocyanate (FITC)-conjugated anti-HLA-DR antibody (clone Immu-357) and anti-CD14-PC5 antibody (clone RMO52) for 10 min at room temperature.

Isotype control antibodies were used to assess nonspecific binding. Lysis of erythrocytes and fixation of cells were performed with FACS lysis solution (BD Pharmingen, San Diego, CA) according to the manufacturer’s instructions. After lysis, cells were washed once with cold PBS, and fluorescence was analyzed on a FACSCanto II flow cytometer (Becton Dickinson) using FACSDiva software (BD Biosciences) as described previously [13]. Data on lymphocytes (10000 events) or monocytes (2000 events) were acquired by gating according to forward and side scatter. Mean fluorescence intensity was converted into antibodies per cell (AB/C) and the FITC signal was calibrated with QIFKIT FITC beads (Dako, CA) according to the manufacturer’s instructions.

### CMV DNA assay

CMV DNA was quantified in stored plasma samples by using the Primer Design Genesig real-time PCR kit (Primer Design Ltd., Chandler’s Ford, UK). DNA was extracted from 200 μl of plasma with a QIAmp DNA mini kit (Qiagen Inc. Valencia, CA), after which the DNA was eluted with 100 μl of 10 mM Tris (pH 8.0) and 10 μl of DNA was used for PCR. A BioRad thermal cycler was employed to perform PCR at 37°C for 15 min and 95°C for 15 min, followed by 50 cycles of 95°C for 10 sec and 60°C for 1 min according to the manufacturer’s instructions. To detect prior CMV infection, CMV IgG was measured with an enzyme immunoassay kit (Medac, Wedel, Germany).

### Statistical analysis

Data were collected in Office Excel 2011 for analysis. All statistical analyses were performed using SigmaPlot software, version 13 (Systat Software Inc., CA, USA). The baseline characteristics were analyzed using Fisher’s exact test for nominal variables and Mann-Whitney rank sum test for continuous variables. Categorical variables are reported as absolute values and percentages, while continuous variables are shown as the median with interquartile range. Contingency table data were compared with Pearson’s chi-square test. Continuous variables were compared among three groups by the nonparametric Kruskal-Wallis test, and p<0.05 was considered to indicate statistical significance.

## Results

A total of 25 patients admitted to ICU were screened initially for CMV IgG. Seven of them were excluded because of a negative result and the remaining 18 patients with a positive anti-CMV IgG titer were enrolled in this study. CMV reactivation was observed in 3 of the 18 patients during the first week of the ICU stay and these 3 patients died (9, 13 and 26 days after admission to ICU).

The median age of the 18 patients was 71 years, with 61% being men and 39% being women. Demographic data and the illness severity scores calculated in the ICU are summarized in Table 1.

**Table 1:** Baseline characteristics of the patients with septic shock and the controls.

Characteristic	Septic shock patients (n = 18) Median(95% CI)	Control subjects (n = 10) Median(95% CI)	*P* value
Age, years	71 (66, 83)	71 (64, 81)	0.34
Male, No. (%)	11 (61)	6 (60)	0.57
Length of ICU stay, days (range)	6.2 (1 to 34)	n/a	
Site of infection, No. (%)		n/a	
Abdomen	8 (44)		-
Respiratory tract	4 (22)		-
Urinary tract	3 (17)		-
Skin/Soft tissue	2 (11)		-
Others	1 (6)		-
Main diagnosis category, No. (%)		n/a	
Medical	8 (44)		
Surgery	10 (56)		
APACHE II score	26 (23, 29)	n/a	
SOFA score	11 (10, 12)	n/a	
Comorbidities		None	
0, No. (%)	7 (39)		-
>1, No. (%)	11 (61)		
Indicators of disease severity, No. (%)		None	
Mechanical ventilation	15 (85)		
Renal replacement therapy	10 (54)		
28-day mortality, No. (%)	3 (17)	n/a	

Abbreviations: APACHE II, Acute Physiology and Chronic Health Evaluation II; SOFA, Sequential Organ Failure Assessment; CI, confidence interval.

The APACHE II score ranges from 0 to 71, with low scores indicating better organ function. The SOFA score ranges from 0 to 24, with lower scores indicating better organ function.

All 18 patients had septic shock, including 10 with disseminated intravascular coagulation and 8 with adult respiratory distress syndrome. Among these 18 patients, 4 had underlying respiratory tract infection, 8 had underlying digestive tract infection, and 3 had underlying urinary tract infection. Gram positive cocci were isolated in 5 patients, gram negative bacilli were isolated in 10 patients, Candida albicans was found in 1 patient, and 2 patient had no confirmed isolate. Seven patients had nosocomial infection.

**Fig 1:**
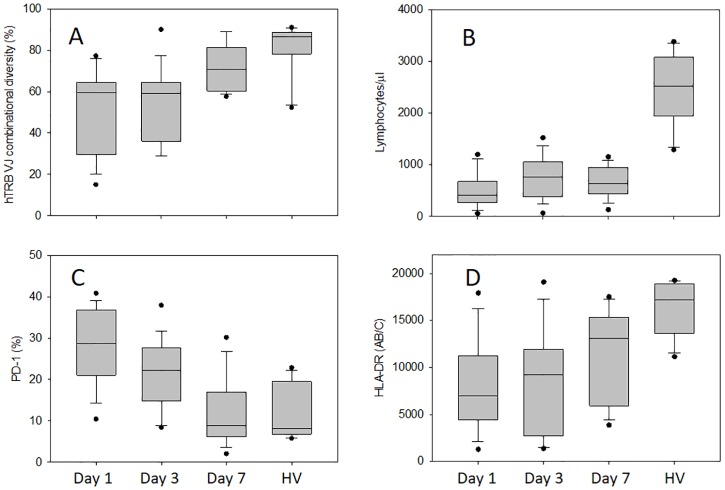
Immune cell surface antigen expression, lymphocyte count, and TCR diversity in septic shock. TCR diversity (expressed as a percentage) was calculated as the ratio of the observed number of rearrangements to the theoretical number (A). Lymphocyte count (B), PD-1 expression by CD4+ T cells (C), and HLA-DR expression by CD14+ monocytes (D) measured on day 1, day 3, and day 7 after the diagnosis of septic shock compared with that in healthy volunteers (HV). Data are shown as box plot with medians (lines inside boxes), 25^th^ and 75^th^ quartiles (lines of boxes) and whiskers indicate the range. Any data not included between the whiskers were plotted as an outlier with small circle. **, p<0.01, *p<0.05 vs. healthy volunteers; ††, p<0.01, †, p<0.05 vs. day 1.

**Fig 2:**
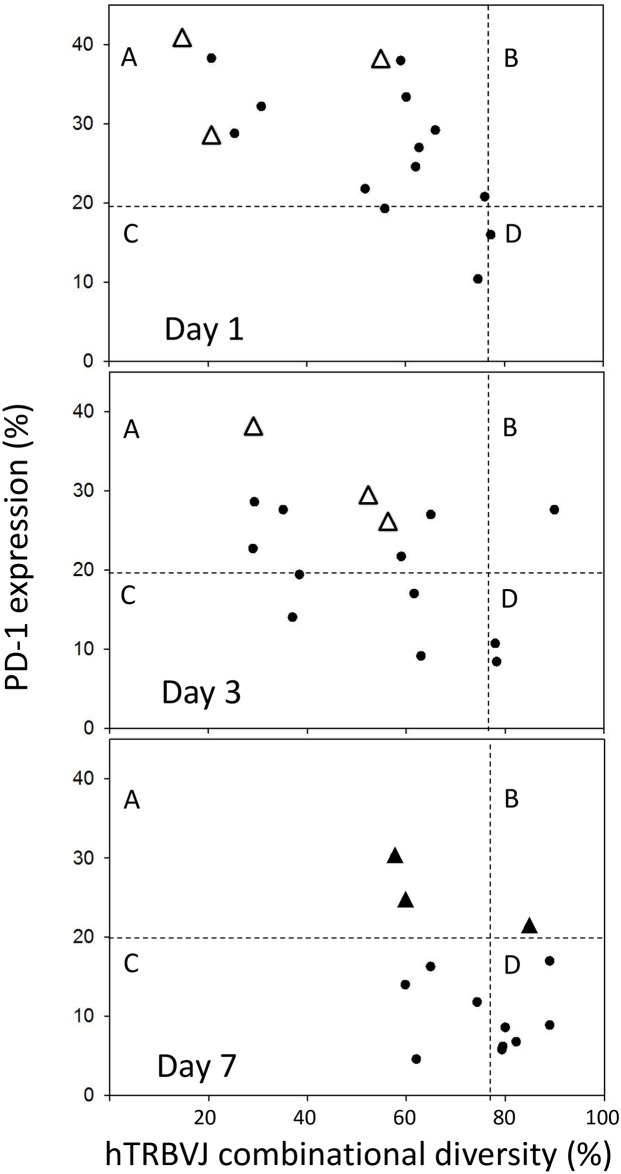
TCR diversity plotted versus PD-1 expression on quadrant charts for days 1, 3, and 7. Each chart is divided into four zones limited by the normal threshold of TCR diversity (78.3% based on lower quantile data from healthy volunteers) and the normal threshold of PD-1 expression (19.5% based on upper quantile data from healthy volunteers). Patients with CMV reactivation who died are indicated by closed triangles on day 7 and by open triangles on days 1 and 3. Group A: high PD-1 expression with low TCR diversity; group B: high PD-1 expression with normal TCR diversity; group C: normal PD-1 expression with low TCR diversity; group D: normal PD-1 expression with normal TCR diversity. Results are presented as individual values.

We used multiplex PCR to investigate the combinatorial diversity of the T cell repertoire. Fig 1 shows the distribution of TCR diversity in the healthy volunteers and in the 18 patients on days 1, 3 and 7 after the diagnosis of septic shock. On day 1, the patients displayed a marked decrease of TCR diversity compared with the healthy volunteers, while recovery of TCR diversity was observed on day 7. The total lymphocyte count was significantly decreased in the patients on days 1, 3, and 7 of septic shock compared with healthy volunteers (Fig 1). We tested the hypothesis that PD-1 expression by T cells plays a key role in lymphocyte dysfunction related to sepsis since we observed marked up-regulation of PD-1 in patients with septic shock (Fig 1). Compared with the healthy volunteers, PD-1 expression by CD4+ T cells was significantly higher in the patients on days 1 and 3 after the diagnosis of septic shock, while normalization of PD-1 expression was observed on day 7, except in the patients who died (Fig 2). In addition, HLA-DR expression was significantly decreased in the patients on days 1, 3 and 7 of septic shock compared with the healthy volunteers (Fig 1).

To investigate whether TCR diversity changed along with PD-1 expression, data on TCR diversity and PD-1 expression obtained at each time point were plotted on quadrant charts (Fig 2). Patients were stratified into the following 4 groups by these parameters on days 1, 3, and 7 using the lower and upper quantile values from healthy volunteers (TCR diversity > 78.3%, PD-1 expression < 19.5% by CD4+ T cells): Group A, high PD-1 expression with low TCR diversity; group B, high PD-1 expression with normal TCR diversity; Group C, normal PD-1 expression with low TCR diversity; and group D, normal PD-1 expression with normal TCR diversity. On day 1, the majority of the patients were in group A, with group C accounting for 13% of the subjects and group D for only 6%. On day 3, there was an increase of patients in group D. By day 7, the majority of the patients were in group D, while group C accounted for 31% and group A for only 15%. Rapid recovery of both TCR diversity and lymphocyte PD-1 expression were observed in the surviving patients (closed circles, p = 0.025, Pearson’s chi-square test), and CMV reactivation did not occur in the survivors. On the other hand, the patients with CMV reactivation who died (closed triangle) still showed T cell abnormalities on day 7 (p = 0.007, Pearson’s chi-square test). On days 1 and 3, none of the patients showed CMV reactivation, but reactivation was observed on day 7. In Fig 2, the three patients with CMV reactivation who died are indicated by open triangles on days 1 and 3, and are indicated by closed triangles on day 7. All three patients were in group A on days 1 and 3.

## Discussion

Immune dysregulation associated with sepsis has been attributed to various mechanisms, and the complex alterations of the immune system in this condition are still not completely understood although several functional defects of adaptive immunity have been characterized in septic patients. The changes that have been recognized include reduction of HLA-DR expression by monocytes [14–16] and increased expression of inhibitory receptors by lymphocytes, including PD-1 [17–20], cytosolic T lymphocyte antigen-4 (CTLA-4) [21], and B and T lymphocyte attenuator (BTLA) [22]. Decreased HLA-DR expression leads to alterations of antigen-presenting function, while increased expression of inhibitory receptors plays a role in lymphocyte exhaustion [8].

In this study, we demonstrated that patients with septic shock show prominent changes of T cell function, in addition to diminished HLA-DR expression on monocytes. We observed a reduction of TCR diversity and increased PD-1 expression by T cells in the early stage of septic shock. Persistent lymphopenia was also observed. Antigen-presenting cells present antigens to lymphocytes in association with major histocompatibility complex II molecules, such as HLA-DR. This is a crucial step in the development of a sustained adaptive immune response that can clear pathogens from the host [23]. The decrease of monocyte HLA-DR expression observed in the present study was consistent with previous reports about rapid and profound down-regulation of HLA-DR expression in patients with septic shock, and this change of HLA-DR status has been proposed as a prognostic marker for shock patients [14–16, 24].

Recently, there have been several reports suggesting that PD-1 may have an important role in sepsis [9, 17–21, 25]. PD-1 is a receptor expressed by lymphocytes and macrophages, and binding of its ligands (PD-L1/PD-L2) inhibits many T cell functions, including cytokine production and cytotoxic activity [26, 27]. In both animals [17, 26, 28] and humans [26, 29, 30], PD-1 has been implicated in the immune suppression that is often associated with cancer and chronic viral infections. In particular, there is experimental evidence of an important role for PD-1 signaling in lymphocyte exhaustion [27, 31, 32]. Blocking the PD-1 pathway improves survival in a mouse model of sepsis, and PD-1 overexpression by circulating T cells has been detected in septic patients and is correlated with a worse outcome [17–19, 33, 34]. In the present study, PD-1 expression on CD4+ T cells was significantly higher than the level in healthy controls on days 1 and 3 of septic shock, and we detected a simultaneous decrease of TCR diversity in the early stage of septic shock. At the early stage, the majority of patients belonged to group A in our quadrant chart classification of lymphocyte function. Some authors have reported that depression of T cell function in patients with sepsis is directly associated with organ failure and increased mortality. The importance of normal lymphocyte function for recovery from septic shock was supported by our observation that the lack of restoration of lymphocyte function (failure to move from group A to group D) was associated with unfavorable outcomes.

Maintenance of lymphocyte function, including a diverse TCR repertoire and an appropriate level of PD-1 expression, is important for the immune system to protect the host against pathogens with novel antigens [25]. Failure of these defense mechanisms impairs the ability of the immune system to mount an appropriate response against invading pathogens and latent viruses such as CMV. The 23% incidence of CMV reactivation in our patients with septic shock is similar to other reports [4, 6]. The CMV reactivation observed in our patients was presumably due to impaired lymphocyte function because these patients did not show progression from group A to group D. Previous studies have demonstrated that chronic HIV infection can lead to defects of virus-specific CD8 T cell effector functions (e.g., cytokine production and/or cytotoxicity) [35]. Thus, it seems possible that viral reactivation in patients with sepsis leads to lymphocyte exhaustion, which further impairs host immunity and results in additional viral reactivation. The persistent lymphopenia observed in this study would lead to suppression of host immunity [36]. However, it is unclear whether the elevated CMV load following reactivation impairs lymphocyte function or whether viral reactivation is more likely to occur in patients with severe sepsis and profound immunosuppression.

In this study, we showed that impairment of lymphocyte function occurs in the early stage of sepsis, which is contrary to the concept that the anti-inflammatory response syndrome is delayed relative to the systemic inflammatory response syndrome. In fact, it is likely that induction of both responses occurs simultaneously, since Osuchowsky and Remick demonstrated that synthesis of both anti-inflammatory and pro-inflammatory cytokines occurred concurrently in an experimental model of sepsis [37].

Little is known about the pattern of recovery of lymphocyte function, including PD-1 expression and TCR diversity, in patients with septic shock. In our study, both TCR diversity and PD-1 expression by lymphocytes were rapidly normalized in the surviving patients. On the other hand, these factors remained abnormal in the patients with a fatal outcome. Condotta et al. reported that septic mice display partial chronic impairment of the available CD8+ T cells repertoire, affecting the host capacity to respond to subsequent infections [38]. On the other hand, Venet et al. reported that a significant decrease of the TCR repertoire in septic patients showed recovery within 1 week, suggesting a role of lymphocytes released from peripheral compartments rather than generation of new lymphocytes by the thymus [39].

A limitation of this study is that we only examined a limited number of patients, which means that our results require validation in a larger sample size. Another limitation is that our age-matched control population consisted of healthy volunteers and not critically ill non-septic patients. Therefore, our findings in the septic patients may have been due to acute critical illness or underlying co-morbidities and not necessarily related to sepsis per se. Furthermore, we could not clarify the effect of lymphocyte depletion on immune function in patients with septic shock.

## Conclusions

We observed reduction of TCR diversity and monocyte expression of HLA-DR along with increased PD-1 expression by CD4+ T cells in the early stage of septic shock. Significant lymphopenia persisted for at least 7 days following the onset of septic shock. Rapid normalization of lymphocyte function occurred in the patients who survived, while persistent lymphocyte dysfunction and CMV reactivation were observed in patients who died. The changes we identified are consistent with the early stage of immune cell exhaustion and our findings emphasize the importance of normal lymphocyte function for recovery from septic shock. Persistence of lymphocyte dysfunction is associated with CMV reactivation/dissemination and with unfavorable outcomes.

## Supporting Information

S1 Fig Examples of an immune repertoire map. Representative examples of TCR diversity for healthy volunteer (S1A) and septic shock patient on days 1 (S1B), 3 (S1C), and 7 (S1D) after the onset of shock. Level of diversity was expressed as percentage through the ratio of observed versus theoretical rearrangements (a). Ten major rearrangements listed by decreasing order of individual contribution to the global repertoire (b). Individual contribution is calculated based on the ratio between individual rearrangement intensity and sum of all rearrangements intensities (c). Each peak represents a rearrangement between a given V gene family and J segment (d).
(TIF)

S2 Fig PD-1 expression on CD4+ lymphocytes. PD-1 expression on CD4+ lymphocytes in healthy volunteer (S2A) and septic shock patient on days 1, 3, and 7 after the onset of shock (S2B). Representative flow cytometric findings are shown.
(TIF)

S3 Fig HLA-DR expression on CD14+ monocytes. HLA-DR expression on CD14+ monocytes in healthy volunteer (SA) and septic shock patient on days 1, 3, and 7 after the onset of shock (S3B). Representative flow cytometric findings are shown.
(TIF)

S4 Fig Quantitative determination of cell surface antigen by flow cytometry. The gate has been set to collect calibration beads signals (a). Histogram of QIFIKIT calibration beads (P2, P3, P4, P5, and P6) populations (b), the MFI of each bead population of the calibration beads (c), and calibration curve (d) are shown. Calibration beads were coated with well-defined quantities of monoclonal antibody.
(TIF)
