# Supplementary figures and images for: Increased PD-1 Expression and Altered T Cell Repertoire Diversity Predict Mortality in Patients with Septic Shock: A Preliminary Study
Source: PLoS One. 2017 Jan 10;12(1):e0169653. doi: 10.1371/journal.pone.0169653 (PMC5225000; doi:10.1371/journal.pone.0169653)

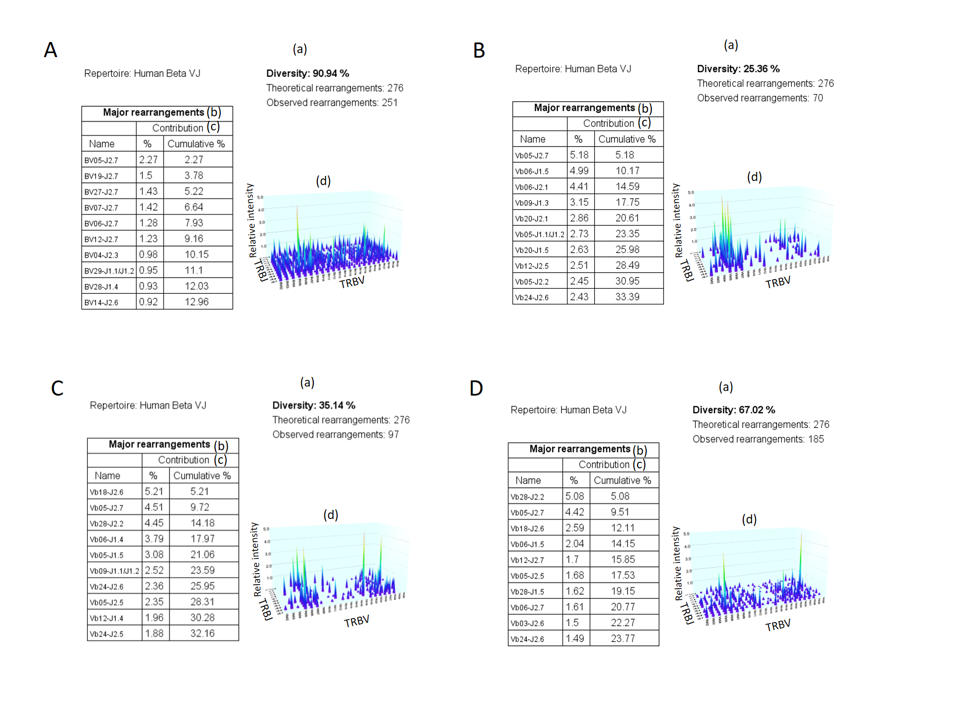

Supplement: S1 Fig — Representative examples of TCR diversity for healthy volunteer (S1A) and septic shock patient on days 1 (S1B), 3 (S1C), and 7 (S1D) after the onset of shock. Level of diversity was expressed as percentage through the ratio of observed versus theoretical rearrangements (a). Ten major rearrangements listed by decreasing order of individual contribution to the global repertoire (b). Individual contribution is calculated based on the ratio between individual rearrangement intensity and sum of all rearrangements intensities (c). Each peak represents a rearrangement between a given V gene family and J segment (d). (TIF) [file pone.0169653.s001.tif]

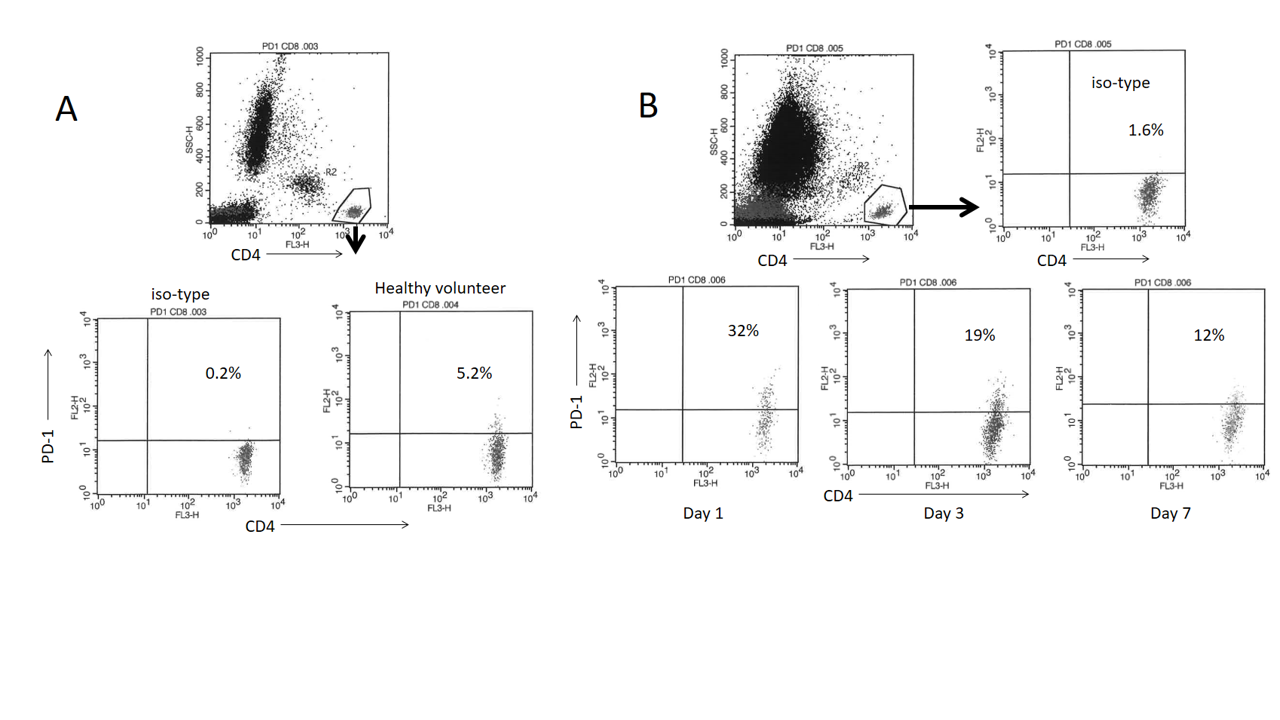

Supplement: S2 Fig — PD-1 expression on CD4+ lymphocytes in healthy volunteer (S2A) and septic shock patient on days 1, 3, and 7 after the onset of shock (S2B). Representative flow cytometric findings are shown. (TIF) [file pone.0169653.s002.TIF]

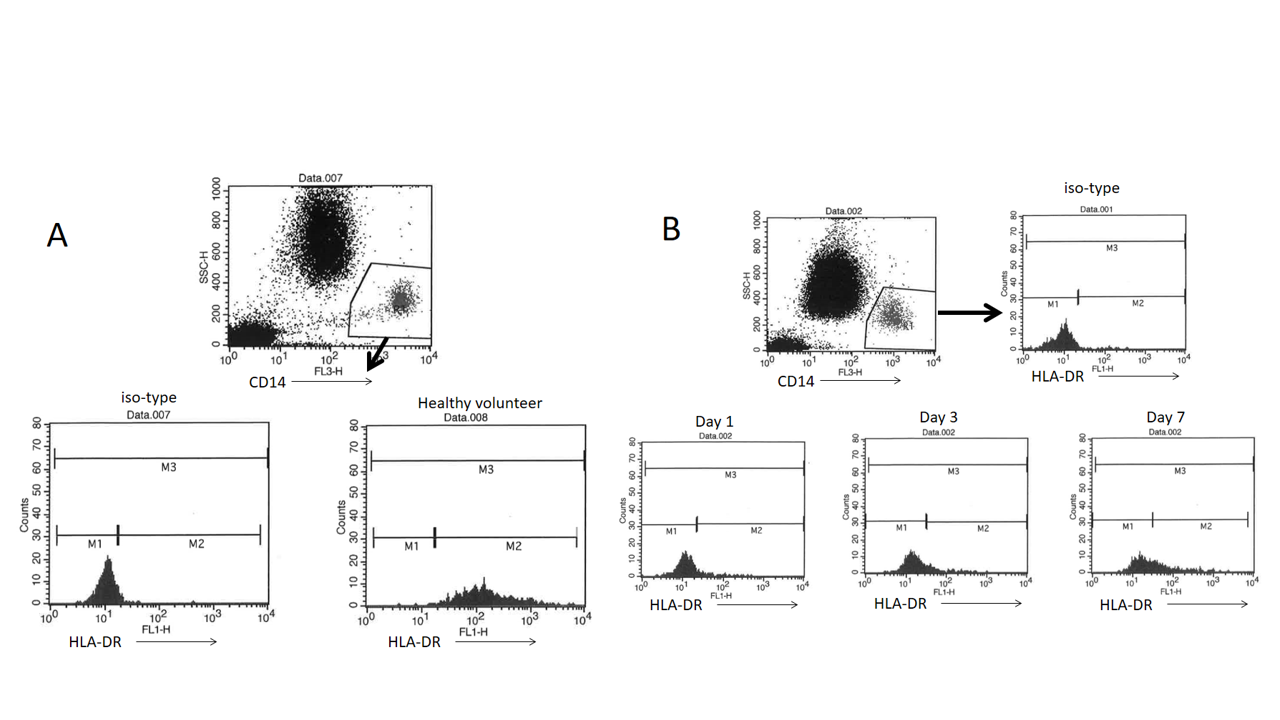

Supplement: S3 Fig — HLA-DR expression on CD14+ monocytes in healthy volunteer (SA) and septic shock patient on days 1, 3, and 7 after the onset of shock (S3B). Representative flow cytometric findings are shown. (TIF) [file pone.0169653.s003.TIF]

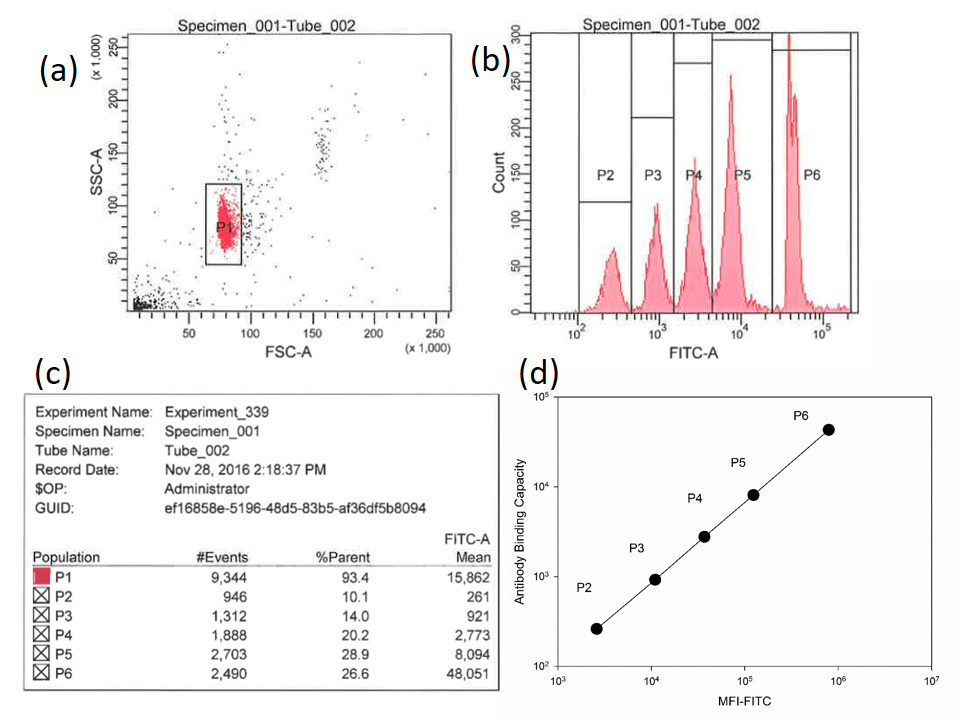

Supplement: S4 Fig — The gate has been set to collect calibration beads signals (a). Histogram of QIFIKIT calibration beads (P2, P3, P4, P5, and P6) populations (b), the MFI of each bead population of the calibration beads (c), and calibration curve (d) are shown. Calibration beads were coated with well-defined quantities of monoclonal antibody. (TIF) [file pone.0169653.s004.tif]
